# How Is Sentence Processing Affected by External Semantic and Syntactic Information? Evidence from Event-Related Potentials

**DOI:** 10.1371/journal.pone.0009742

**Published:** 2010-03-17

**Authors:** Annekathrin Schacht, Manuel Martín-Loeches, Pilar Casado, Rasha Abdel Rahman, Alejandra Sel, Werner Sommer

**Affiliations:** 1 Department of Psychology, Humboldt-Universitaet zu Berlin, Berlin, Germany; 2 Center for Human Evolution and Behavior, UCM-ISCIII, Madrid, Spain; 3 Psychobiology Department, Complutense University of Madrid, Madrid, Spain; Kyushu University, Japan

## Abstract

**Background:**

A crucial question for understanding sentence comprehension is the openness of syntactic and semantic processes for other sources of information. Using event-related potentials in a dual task paradigm, we had previously found that sentence processing takes into consideration task relevant sentence-external semantic but not syntactic information. In that study, internal and external information both varied within the same linguistic domain—either semantic or syntactic. Here we investigated whether across-domain sentence-external information would impact within-sentence processing.

**Methodology:**

In one condition, adjectives within visually presented sentences of the structure [Det]-[Noun]-[Adjective]-[Verb] were semantically correct or incorrect. Simultaneously with the noun, auditory adjectives were presented that morphosyntactically matched or mismatched the visual adjectives with respect to gender.

**Findings:**

As expected, semantic violations within the sentence elicited N400 and P600 components in the ERP. However, these components were not modulated by syntactic matching of the sentence-external auditory adjective. In a second condition, syntactic within-sentence correctness-variations were combined with semantic matching variations between the auditory and the visual adjective. Here, syntactic within-sentence violations elicited a LAN and a P600 that did not interact with semantic matching of the auditory adjective. However, semantic mismatching of the latter elicited a frontocentral positivity, presumably related to an increase in discourse level complexity.

**Conclusion:**

The current findings underscore the open versus algorithmic nature of semantic and syntactic processing, respectively, during sentence comprehension.

## Introduction

Undisputedly, full comprehension of sentences requires combining the meaning of individual words with syntactic structure. Yet the nature of semantic and syntactic processing and their confluence remain controversial. Because they provide relatively direct and specific indicators of these processing streams, event-related brain potentials (ERPs) have frequently been employed to study the properties and interplay of semantic and syntactic processing. Although ERPs have provided valuable, albeit incomplete, evidence about the properties of the semantic and syntactic processing streams, when their confluence and interplay is concerned, the evidence is heterogeneous (see [1]). In the present study, we aimed at specifying the characteristics of the syntactic and semantic processing streams and their interaction by combining sentence processing with processing sentence-extraneous linguistic material.

The interplay between different processing systems depends on the degree of openness of the systems; the more open and cognitively penetrable a process, the higher the chances for interactions. Strongly modular models assume that informationally encapsulated and at least partly sequential processes construct distinct syntactic and semantic representations [2]–[3]. In contrast, fully interactive models suggest that syntactic and semantic constraints interact directly and simultaneously with each other at the message-level representation of the input [4]–[6]. Other intermediate proposals differ in the degree of independence and prevalence ascribed to conceptual/semantic and syntactic information (e.g., [7]–[9]).

A widely used methodological approach to questions concerning the properties and interplay of semantic and syntactic processes is the recording of ERPs, which permit on-line measurements of electrical brain activities as language processing unfolds over time. Indeed, different ERP components support a distinction between the processing of syntactic and semantic information. When semantic variables are manipulated, the main finding is the so-called N400 effect [10], a negative-going ERP component, which is usually most pronounced between roughly 250 and 550 ms after word onset, with a maximum over central and posterior electrode sites [11]. This component typically increases in amplitude with the difficulty of integrating words into their semantic context, provided by a sentence or a preceding prime word [12]. When syntactic variables are manipulated, the main ERP effects are anterior negativities and posterior positivities. Anterior negativities are typically labeled as LAN (left anterior negativity) and resemble the N400 in latency, though a so-called ELAN (early LAN) may appear as early as 100 to 200 ms after word onset. Word category violations are the variables most frequently associated with ELAN (e.g., [13]), whereas other grammatical anomalies, including morphosyntactic violations (e.g., [14]), usually evoke a LAN. Both types of anterior negativities are suggested to reflect highly automatic first-pass parsing processes, the detection of a morphosyntactic mismatch, and/or the inability to assign the incoming word to the current phrase structure [15].

A late positive-going component with a parietal maximum, labeled P600, has typically been considered as another syntax-related ERP fluctuation [16], mainly because it is elicited by syntactic violations (e.g., [17]) and by structurally ambiguous – garden path – sentences (e.g., [18]). The P600 has been suggested to reflect increased syntactic processing costs due to necessary revisions and re-analyses of structural mismatches and, possibly, subsequent repair processes [19]. Recent observations of P600 deflections to purely semantic violations ([20] for a review) have motivated alternative proposals. Accordingly, the P600 might reflect the activity of a combinatorial system that integrates both semantic and syntactic information, but this system would still be syntactic in nature because its main function would be the assignment of thematic roles [21]. Another suggestion is that the P600 reflects a domain-general monitoring mechanism [20].

### Within-sentence interplay of semantics and syntax

In order to study the implementation of semantic and syntactic constraints during sentence processing and their interplay, factorial designs have been used, in which semantic and syntactic violations are presented in isolation, combination, or both. As pointed out by Martín-Loeches and co-workers [1], the results from such experiments have been highly heterogeneous. In their own experiment with Spanish sentences, these authors factorially combined syntactic and semantic violations in the same sentence-intermediate adjective. Violations consisted in noun–adjective number or gender disagreements (syntactic violation), noun–adjective semantic incompatibility (semantic violation), or both (combined violation). The N400 to semantic violations was unaffected by additional syntactic violations. In contrast, the P600/SPS component was elicited by both pure syntactic and semantic violations, but seemed to be diminished in combined violations relative to single syntactic violations. The authors suggested that – at least under the conditions of their experiment – semantic information may have a prevailing role over syntactic information. On a more general level, and in line with other reports, the results indicate that semantic and syntactic streams may indeed interact, as evidenced, in particular, in the P600 component. However, considering the long latency of the P600 on the one hand and the speed of sentence comprehension on the other hand, one wonders whether this component should really be the earliest sign of semantics/syntax interplay.

### The specific nature of syntactic and semantic processing

The possibility of any interaction or interplay between different processes depends on their properties. Encapsulated modules would be non-interactive by definition. In this case, any effects on the different processes would be additive. On the other hand, if the processes are non-modular and open or penetrable to extraneous information, they might be affected by a common experimental factor. For example, semantic processing might be directly affected by syntactic variables if the semantic system is open to syntactic information. On the other hand, if two processes are modular and impenetrable to other kinds of information, only the end-products of each process could converge in a third kind of processing stream. Now, what is the status of syntactic and semantic processes in terms of their openness or modularity?

The syntactic stream has been considered as algorithmic, following a finite list of well-defined rules, governing how words and other lexical elements combine to form phrases and sentences [22]. In contrast, the semantic stream presents itself as an open system, where sentences are treated as unordered lists of words that are combined on the basis of plausibility according to our personal and flexible world-knowledge [23]–[24]. It has been suggested that semantic information during sentence processing should be subdivided into associative memory-based semantic relationships on the one hand and semantic-thematic relationships on the other hand, which – in turn – have implications for sentence structure [21]. The open and flexible nature of associative memory-based semantic constituents appears to be obvious, but the same cannot be said for semantic-thematic relationships. On the other hand, it is also possible that the syntactic system is not totally algorithmic. In this regard, several authors have stressed the relevance of some heuristics in sentence comprehension, such as word order, that appear to be syntactic in nature [23], [25]–[26].

Recent advances in neurosciences appear to substantiate the validity of the two main systems proposed by psycholinguists as neuroanatomically segregated streams. As a plausible scenario, the syntactic stream appears to involve a dorsal pathway comprising parietal, superior temporal and premotor inferior frontal regions, connected via the arcuate fasciculus. In contrast, the semantic stream apparently involves a more ventral system, comprising middle temporal and ventrolateral prefrontal cortex, connected via different neural pathways [27]–[29]. Although this overall scheme of anatomically segregated pathways is still incomplete, it is in line with qualitatively different functional properties of the semantic and syntactic streams.

From these considerations above, it would seem that the options for any interactions between semantics and syntax are limited because although semantics appears to be rather open and non-modular, this seems to hold much less for syntax. But it has to be stated that the range of experimental variables, exploring the properties of language processing streams, is far from being exhausted. Hitherto mainly interactions within a given sentence have been studied, that is, all variables of interest have been manipulated within the same information source – the sentence. However, for a full characterization of the properties of linguistic information streams and their interaction it would be important to know whether they are confined to a given sentence structure or cross these boundaries.

### The impact of external linguistic information on semantic and syntactic processes

Recently, the present authors [30] have applied a dual task paradigm to assess whether syntactic and semantic processes within a sentence would be influenced by sentence external information. To this end, written sentences were to be processed while sentence-extraneous spoken material was to be held in working memory. The written sentences (Task 1) could be correct or incorrect from either the syntactic or the semantic point of view, yielding anterior negativities and P600 components in the former case, and an N400 in the latter. Each sentence had to be judged for correctness. Shortly before the violation within the written sentence, which was shown word by word, a sentence-extraneous spoken word was presented. Participants had to keep this word in working memory (Task 2), since they had to repeat it after the end of the sentence.

The spoken words showed specific features that could constitute semantic or syntactic mismatches with respect to the sentences. Whether or not these sentence-extraneous features were integrated into and interacted with sentence processing was assessed by measuring the ERPs elicited by the written words of the sentences. As a main finding, syntactic processing within the sentence appeared to be blind to the syntactic content of the sentence-extraneous material, reflected in a LAN, which was unaffected by mismatches produced by the spoken words. In contrast, semantically mismatching sentence-extraneous material induced ERP fluctuations typically associated with the detection of within-sentence semantic anomalies (N400) even in semantically correct sentences. Subtle but extant differences in topography between this externally induced N400 and the N400 to within-sentence semantic violations added support to recent proposals of separate semantic subsystems, differing in their specificity for sentence structure and computational procedures.

Interestingly, semantic violations also elicited a P600, albeit smaller than the P600 to within-sentence syntactic violations. Strikingly, this P600 was influenced by the semantically mismatching sentence-extraneous material, supporting current proposals that the P600 reflects a third combinatorial stream in sentence comprehension which integrates both semantic and syntactic information.

Together, these findings provided novel evidence for the assumption that the syntactic and semantic processing systems differ in their general properties. While semantic processes appear to be open to context information (cf., [31]), syntactic processing seemed rather encapsulated and immune against such external influences. The findings also give a first impression on how external linguistic information may interfere with sentence-internal analyses.

### The present study

Our previous study had tested the effects of sentence-extraneous material on within-sentence processing only for the same type of information, that is, semantic-semantic and syntactic-syntactic interactions. The purpose of the present study was to explore effects across different types of processes, complementing the results of our previous study. That is, we investigated the influence of sentence-extraneous syntactic information on within-sentence semantic processing, as well as the influence of sentence-extraneous semantic information on within-sentence syntactic processing.

In the present study, the written sentences (Task 1) could be correct or incorrect from either the syntactic or the semantic point of view. Both kinds of violation occurred in a particular word of the sentence. Each sentence had to be judged for correctness following its presentation. As an example, the sentence *Los enemigos*_[masc.]_ *agresivos*_[masc.]_ *luchan* (literally: *The enemies_[masc.]_ aggressive_[masc.]_ fight*) could be violated syntactically by modifying the gender of the adjective (*agresivas*_[fem.]_), and semantically by replacing the correct adjective by an inappropriate one (*opacos*_[masc.]_ = *opaque*_[masc.]_).

Shortly before the violation within the sentence, a single spoken word (an adjective) was presented. Participants had to keep this word in working memory (Task 2), since they had to repeat it after giving correctness judgments about the sentence. In the syntactic condition, morphosyntactic violations (gender agreement violation in Spanish) within the written sentence were preceded by spoken adjectives that either semantically matched (*coléricos*_[masc.]_ = *furious*_[masc.]_) or mismatched (*velados*_[masc.]_ = *fogged*_[masc.]_) the violation in the sentence. In the semantic condition, semantic violations of the adjectives within the sentence were preceded by spoken adjectives that syntactically matched (*velados*_[masc.]_ = *fogged*_[masc.]_) or mismatched (*veladas*_[fem.]_ = *fogged*_[fem.]_) the written adjective. For correct sentence material, the spoken adjectives could also syntactically or semantically match or mismatch. This procedure provides a dual task paradigm that to a large extent resembles the circumstances concurring in the Reading Span Test [32] delivered to study linguistic working memory capacity. In the most standard version of this test, participants must read several sentences for comprehension while simultaneously keeping the last word of each sentence in working memory. Despite discrepancies on the working memory system or subsystem involved by this test [32]–[33], there is consensus that in the Reading Span Test the last word of each sentence is kept within the same working memory system or subsystem where sentence comprehension takes place, thus, disturbing the latter and vice versa.

### Predictions

For *within-sentence violations* we expected the usual N400 and P600 components to semantically incorrect relative to correct adjectives and a LAN and a P600 to syntactically incorrect relative to correct adjectives. For correct sentences, semantic mismatches of sentence-external adjectives should elicit an N400 and a (semantic) P600 – confirming our previous findings [30] and being in line with the assumption of a flexible and open nature of semantic processing. In contrast, due to the algorithmic nature of syntactic processing, extraneous syntactic mismatches should not elicit a LAN or a (syntactic) P600 in correct sentences.

As regards the *interplay* between semantic and syntactic processes across the sentence context, we expected a differential modulation of the (syntactical) P600 by sentence external semantically matching and mismatching material. This prediction was based (a) on the suggestion that the semantic system is open and takes in information from multiple sources [30] and (b) on reports about semantics-syntax interactions in the P600 amplitude due to purely sentence-internal double violations (for review see [1]). In contrast, we did not expect a modulation of the N400 or P600 components in semantically incorrect sentences due to syntactically matching or mismatching sentence-external information. This is because we expected no intrusion of syntactical sentence external material into sentence processing, precluding any interaction.

## Methods

### Participants

Participants were 32 native Spanish-speakers (26 females, mean age 25.3 years, range 17–51). All were right-handed, with average handedness scores of +82, ranging from +33 to +100, according to the Edinburgh Handedness Inventory [34]. The study was performed in accordance with the Declaration of Helsinki, and approved by the ethics committee of the Center for Human Evolution and Behavior, UCM-ISCIII, Madrid, Spain. Participants gave written consent to the study and were reimbursed thereafter.

### Materials

Table 1 gives examples for the experimental materials. The set of items for the sentence processing task (Task 1) was based on 160 Spanish *correct sentences* from each of which a semantically and a syntactically incorrect version was derived. All sentences had the structure, [Det]-[N]-[Adj]-[V] (determiner-noun-adjective-verb). In these materials, all nouns and adjectives are marked for gender. The first incorrect sentence version contained a *semantic violation* due to an unacceptable combination of noun and adjective. The second incorrect version contained a *syntactic violation* of the gender agreement between noun and adjective by modifying the latter. In all versions of the sentences, the critical words (the adjectives) were of comparable familiarity (19 per million), according to the “Lexico Informatizado del Español” (LEXESP [35]), and number of letters (*M*s = 7.4, for correct and syntactically anomalous adjectives, and 7.5 for the semantically anomalous adjectives).

**Table 1:** Examples of stimulus materials with word-by-word translations into English and nonliteral interpretations.

Condition	Example	
	Match^1^	Mismatch^1^
**Semantic Condition**		
**Correct** Sentence	La fiesta_[fem.]_ lujosa_[fem.]_ empieza.	
	The party_[fem.]_ luxurious_[fem.]_ starts. ( = *The luxurious party starts*)	
	casada_[fem.]_/married_[fem.]_ 2	casado_[masc.]_/married_[masc.]_
**Incorrect** Sentence	La fiesta_[fem.]_ casada_[fem.]_ empieza.	
	The party_[fem.]_ married_[fem.]_ starts. ( = *The married party starts*)	
	rugosa_[fem.]_/wrinkly _[fem.]_	rugoso_[masc.]_/wrinkly_[masc.]_
**Syntactic Condition**		
**Correct** Sentence	La fiesta_[fem.]_ lujosa_[fem.]_ empieza.	
	The party_[fem.]_ luxurious_[fem.]_ starts. ( = *The luxurious party starts*)	
	pomposo_[masc.]_/pretentious_[masc.]_ 3	casado_[masc.]_/married_[masc.]_
**Incorrect** Sentence	La fiesta_[fem.]_ lujoso_[masc.]_ empieza.	
	The party_[fem.]_ luxurious_[masc.]_ starts. ( = *The luxurious party starts*)	
	pomposa_[fem.]_/pretentious_[fem.]_	casada_[fem.]_/married_[fem.]_

Underlined is the critical word (adjective) in the visually presented sentence.

1 Morphosyntactic or semantic matches and mismatches relationship the acoustic adjective given here and the critical adjective in the visually presented sentence.

2 Morphosyntacic matches and mismatches.

3 Semantic matches and mismatches.

Materials for the acoustic *memory task* (***Task 2***), consisted in a set of adjectives, constructed according to the following principles (cf. Table 1). In the semantic condition, half of these acoustic adjectives were *syntactically matching*, the other half *syntactically mismatching* to both the visually presented noun and adjective of one of the sentences of Task 1. All acoustic adjectives in this condition were semantically mismatching to both the noun and adjective of the sentence.

In the syntactic condition, acoustic adjectives were either *semantically matching* or *mismatching* to both noun and adjective of a given sentence of Task 1. In terms of syntactic relations, half of these adjectives were matching, the other half mismatching to the visual noun and were always mismatching to the critical adjective of the sentence. These principles were applied in order to achieve appropriate combinations of Task 1 vs. Task 2 adjectives, as described below in the Procedure section.

In addition to the experimental sentences, a set of 160 filler sentences was constructed. Half of them (short fillers) followed the same structure as the experimental materials but the adjective was omitted. For the remaining fillers (long fillers), a complement was appended to the structure of the experimental sentences. Half of both short and long fillers were unacceptable sentences, with syntactic or semantic violations - depending on condition - either in the verb or in the complement, for long and short fillers, respectively. Acoustic adjectives for the filler sentences were constructed according to the same procedure as used for the experimental sentences.

The full set of experimental items involved 160 each of correct and semantically and syntactically incorrect sentences, each sentence being combined with a matching or mismatching acoustic adjective. The correct sentences with their corresponding acoustic adjectives were then doubled. Thus, there was a total of 1280 experimental sentences (640 correct sentences and 320 each semantically and syntactically incorrect ones). These 1280 sentences were subdivided into four subsets of 320 sentences, where each condition combination of the factors correctness (correct vs. incorrect), condition (semantics vs. syntax), and matching (matching vs. mismatching) was represented by 40 sentences. None of the 320 experimental sentences (plus acoustic adjective) within a given subset of materials was repeated. A given participant was presented with one of these subsets of 320 experimental sentences plus 320 filler sentences (plus acoustic adjectives). For all participants alike, filler sentences consisted of the 160 filler items described above, repeated once in the second half of the experiment.

All stimuli for the sentence processing task were matched in visual aspects and presented white-on-black on a computer monitor, controlled by Presentation® Software at a viewing distance of 65 cm, resulting in stimulus size of 0.7° to 1.3° height, and 1.1° to 6° width. All adjectives in Task 2 were comparable in intensity and voice of speaker and were presented by means of loudspeakers located in front of the subjects. Overall intensity levels of the acoustic adjectives were adjusted to a comfortable listening level for each participant.

### Procedure

The whole experimental session took about 90 minutes. Participants performed eight experimental blocks each consisting of 40 experimental sentences, 20 short, and 20 long fillers, resulting in 40 experimental sentences per condition on the whole. Importantly, sentences of all possible conditions, that is semantically and syntactically correct, semantically incorrect, syntactically incorrect, and filler sentences, were presented randomly within the experiment. Within a block none of the experimental sentences of a given set was repeated.

Participants performed both tasks simultaneously. In ***Task 1***, participants had to judge each sentence for correctness, i.e., whether it is an acceptable sentence of Spanish or not, by pressing one of two buttons as soon as they detected an unacceptable word, or just after the last word for correct sentences. Correctness judgments were given with index fingers. The assignment of hand to response type was counterbalanced. All sentences began with a fixation cross of 500 ms duration and were presented word-by-word, with 300 ms duration per word and a 600-ms SOA, allowing 4.3 s between the end of the last word in a sentence and the appearance of the first word in the next sentence. The first word in each sentence began with a capital letter and the last word ended with a period.

**Figure 1:**
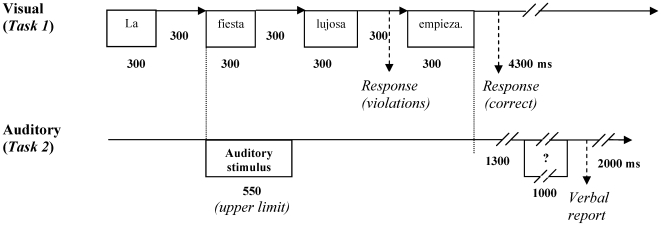
Schematic representation of the stimulation procedures. Two tasks were used simultaneously: Read a sentence presented word by word and judge for acceptability (Task 1), and hear a word, retain it, and say it aloud after the end of the sentence in write (Task 2).

***Task 2*** required participants to keep the acoustic adjective in mind and to repeat it after the end of the written sentence, including the specific gender information. A question mark appeared on the screen for 1 s, starting 1.3 s after the last word of the sentence, prompting the repetition of the spoken adjective. Spoken word duration was variable but not longer than 550 ms for adjectives co-occurring with the experimental visual sentences of Task 1. Spoken adjectives co-occurring with filler sentences could have longer durations. The onset of the spoken word was always synchronized to the onset of the noun in the visual sentence. A scheme of the structure of an experimental trial is represented in Figure 1.

**Figure 2:**
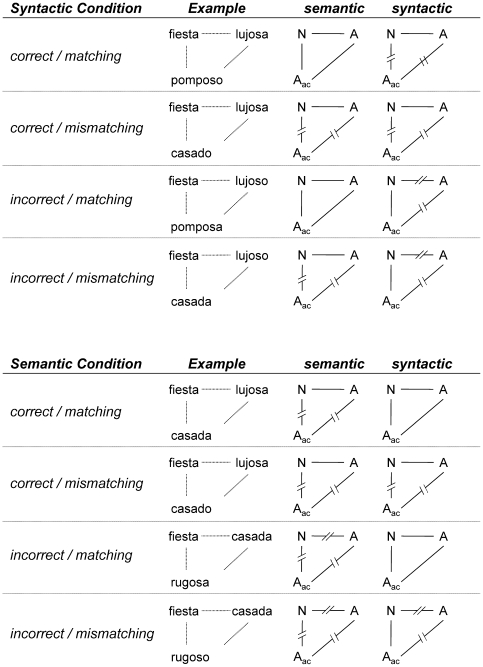
Overview about experimental conditions. Schematic overview about conditions and their resulting semantic and syntactic relations between the visual noun and adjective of Task 1 and between the acoustic adjective of Task 2 and the both visual noun and adjective.

As becomes clear from Figure 1, the experimental manipulation of sentence correctness and matching between the acoustic and the visual adjective also has consequences for the relationship between the spoken adjective and the written noun. An overview of these relationships is given in Figure 2. The basic idea for this experiment was to have a semantic or syntactic manipulation within a sentence (Task 1) and to study how the within-sentence processing is affected by extraneous linguistic information of the other type in Task 2. That is, semantic correctness manipulations within the sentences of Task 1 were combined with syntactic matching manipulations between this within-sentence stream and the extraneous adjectives of Task 2, while syntactic correctness manipulations within the sentences of Task 1 were combined with semantic matching manipulations between both streams. In consequence, it is impossible to keep both semantic and syntactic relationships between simultaneously presented acoustic and visual adjectives at a fixed level. Thus, in the semantic condition the syntactic match between the noun and the auditory adjective will covary with the matching of the two adjectives in a reversed way. In the syntactic condition, the syntactic relations between the noun and the auditory adjective will vary with syntactic correctness of the sentence and the semantic relationship will covary with the matching manipulation between both adjectives. Figure 2 shows these unavoidable complications. As a necessary consequence, several types of combinations of sentences with extraneous material appeared twice as often as other combinations. Nevertheless, we would like to point out that although the relationships between visual noun and auditory adjective may covary, the linguistic relationships (violations and mismatches) relative to the target word (the visual adjective) are factorially combined and therefore represent independent experimental factors in both semantic and syntactic conditions.

### Electrophysiological recording and analysis

The electroencephalogram (EEG) was recorded from 27 tin electrodes mounted within an electrode cap (ElectroCap International). All electrodes were referenced online to the right mastoid, and re-referenced offline to the average of the left and right mastoids. Bipolar horizontal and vertical electrooculograms (EOG) were recorded for artifact monitoring. At the beginning of the experiment, electrode impedances were typically below 3 kΩ. The signals were recorded continuously with a band-pass from 0.01 to 30 Hz and a sampling rate of 250 Hz.

Offline, the continuous EEG was segmented into 1800-ms epochs starting 200 ms before the onset of the visual noun in the experimental sentences. Artifacts were automatically rejected by eliminating epochs during which a range of +100 µV was exceeded in any of the channels. Corrections for artefacts due to blinks and vertical or horizontal eye movements were made using the method described by Gratton, Coles, and Donchin [36]. Based on visual inspection, all epochs that still contained artefacts after automatic rejection were eliminated. Epochs with erroneous judgments or responses (correct sentences judged as unacceptable, incorrect sentences judged as acceptable, or incorrect verbal reports in Task 2) were also eliminated. Overall, 32.7% of the trials had to be discarded: 3.5% due to artefacts, 13.9% because of incorrect responses in Task 1, and 15.3% due to errors in Task 2. The mean number of remaining trials for each condition ranged between 25 and 29.

Separate ERP waves were analyzed for epochs containing adjectives in the experimental sentences as a function of whether they were correct or not and preceded by an auditory adjective in Task 2 matching or mismatching syntactically or semantically, separately for each corresponding condition (syntactic, semantic). Comparisons involved main effects of within-sentence correctness, main effects of sentence extraneous matching, as well as their interaction.

Following the procedure used in our previous study [30], we aimed to analyze the ERPs using a baseline in a 100-ms segment before the onset of the critical (visual) adjective. However, as mentioned above, in the present study experimental manipulations might induce effects between the simultaneously presented acoustic adjectives and visual nouns, that is, prior to the onset of the critical adjectives. Therefore, in a first step the ERP effects of the experimental manipulations (factors correctness and matching) were investigated within this baseline interval (100 ms before the onset of the visual adjective) while applying a baseline of 200 ms before the onset of the noun and the acoustic adjective. In case of non-significant effects within this interval, the more proper baseline, time-locked to the onset of the critical word (the adjective), would become valid. In contrast, in case of significant effects a pre-noun baseline would be better suited even for the fluctuations subsequent to the appearance of the adjective.

According to our previous study, we expected the following ERP components to be affected by experimental conditions: In the semantic condition, an N400 should be elicited by violations within the sentences between 350 and 450 ms. Syntactic violations within the visual stream should elicit a LAN between 400 and 500 ms. In both conditions, a P600 can be expected between 700 and 1000 ms. For investigating these components and their modulations by the mis/matching extraneous acoustic information, overall repeated-measures analyses of variance (ANOVAs) were performed on ERP mean amplitudes in consecutive 50 ms time windows within these specified intervals (350–500 ms for LAN and N400; 700–1000 ms for P600).

Repeated measures ANOVAs were calculated with factors Correctness of the sentence, Matching between the acoustic and the visual adjective, and – in case of ERP amplitudes – Electrode site (27 levels). For interactions between experimental conditions and Electrode site, Greenhouse-Geisser correction was applied to adjust degrees of freedom of *F* ratios.

## Results

### Semantic condition

#### Performance

ANOVA on error rates yielded no significant main effects of Correctness, Matching, or an interaction of these factors, all *F*s(1,31)<1 (17.2%≥*M*s≤16.8%). Mean RTs for correct sentences were 1599 and 1602 ms after adjective onset when the acoustic adjective of Task 2 syntactically matched or mismatched, respectively. Mean RTs for incorrect sentences were 1502 and 1503 ms when the auditory adjective of Task 2 matched or mismatched, respectively. An ANOVA yielded the expected significant main effect of Correctness, *F*(1,31) = 9.6, *p*<.01, while neither a main effect of Matching nor an interaction between these factors appeared, *F*s<1. However, this main effect of Correctness is trivial since responses to violations could be given immediately after their occurrence, whereas correctness judgments were justified only at the end of a sentence.

#### ERP data

As described above, in a first step the baseline interval preceding the onset of the visual adjective was tested for experimental effects against the initial baseline prior to the noun's onset. Since the experimental factor Correctness can not affect the relations between the noun and the acoustic adjective, this factor was dropped from the ANOVA on mean ERP amplitudes between 500 and 600 ms. No effect of Matching, *F*(1,31) = 1.2, *p*>.05, or interaction of Matching with Electrode, *F*(26,806)<1, was obtained. Therefore, for the analysis of experimental effects within the intervals following adjective onset, all ERPs were recalculated to a pre-adjective 100 ms-baseline, conforming to our previous report [30].

**Figure 3:**
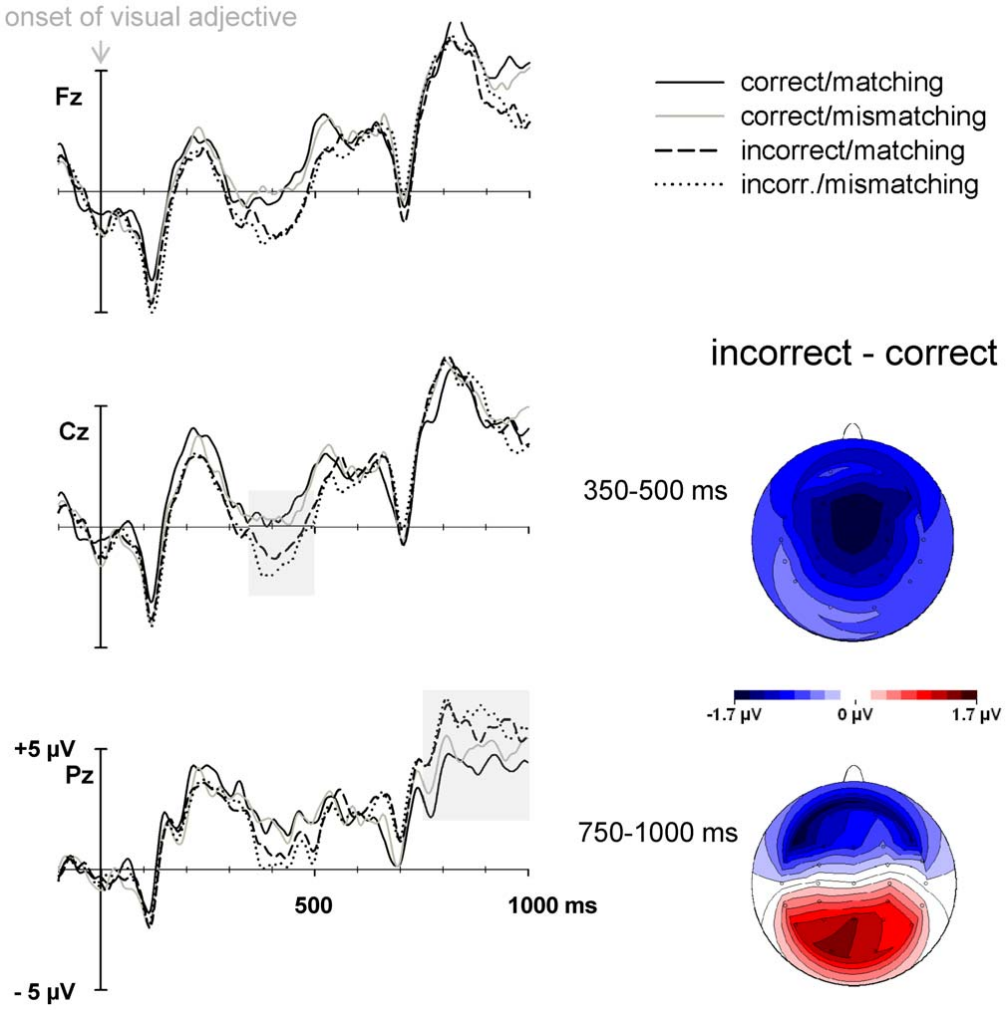
ERPs to semantically correct and incorrect adjectives, referred to a 100 ms pre-noun baseline, as a function of whether they were preceded by an acoustic stimulus matching or mismatching syntactically with the adjective of the sentence. *Left*. ERP waveforms at a selection of electrodes. *Right*. Difference maps of the effects in the N400 and P600 time windows.

Semantic violations (factor Correctness) modulated ERP amplitudes between 350 and 500 ms as main effect, all *F*s(1,31)>10.3, *p*s<.01, and between 350 and 450 ms in interaction with Electrode, *F*s(26,806) = 3.0 and 2.8, *p*s<.05, *ε*s = .160 and .161 (cf. Table 2). Figure 3 depicts main ERP results for the semantic condition, showing the overlays of the ERP waveforms for the adjective in the four conditions. As can be seen also in Figure 3, the Correctness effect consists of an enhanced negativity to semantically incorrect relative to correct sentences and is most pronounced at central and frontocentral electrodes, which is typical for the N400 component to semantic violations. This N400 deflection appeared to be unaffected of whether the auditory adjective of Task 2 preceding the semantic violation was syntactically matching or mismatching to the violating visual adjective, as reflected in the absence of any main effects of Matching, all *F*s<1. In addition, the interaction between Matching and Correctness failed to reach significance, all *F*s<1.

**Table 2:** ANOVA results – semantic condition.

Source	df	350–400	400–450	450–500	700–750	750–800	800–850	850–900	900–950	950–1000
Correctness (Corr.)	1,31	10.3***	15.2***	13.7**						
Matching (Match.)	1,31									
Corr.*Match.	1,31									
Corr.*Electrode (El.)	26,806	3.0*	2.8*			6.6***	9.7***	8.8***	11.8***	12.9***
		.160	.161			.192	.171	.180	.176	.147
Match.*El.	26,806									
Match.*Corr.*El.	26,806									

Note. *F*-values with *p* (***<.001, **<.01, *<.05) and *ε* for Greenhouse-Geisser correction. Only significant results are reported.

During a subsequent time period, and mainly at parietal electrode sites, semantic violation yielded a P600 component that appeared to be similar regardless of the information contained in Task 2. Significant Correctness by Electrode interactions in all consecutive 50-ms segments between 750 and 1000 ms, *F*s(26,806)>6.5, *p*s<.001, .146<*ε*>.193, statistically support this impression. In these time segments, neither main effects of matching nor interactions between any of the experimental factors appeared, all *F*s<1.

In order ensure that individual differences in memory performance did not affect the main ERP findings, we conducted a further analysis. Participants were divided into good and bad performers according to a median split of their overall error rates in Task 2. This variable was added as between-subject factor to the ANOVAs on ERP mean amplitudes of the semantic N400 and the fronto-central P600. Because none of the interactions with correctness or matching approached significance (all *F*s<1), the effects of these factors seem to be unrelated to memory performance.

### Syntactic Condition

#### Performance

In the *syntactic condition*, correct sentences were judged as acceptable in 86.0% of the cases when the auditory adjective of Task 2 syntactically matched with the adjective in the sentence, and in 84.9% of the cases when it mismatched. Incorrect sentences were generally judged more accurately as unacceptable, whether the auditory adjective was semantically matching or mismatching, *M*s = 93.7 and 93.4%, respectively. Thus, acceptability yielded a main effect, *F*(1,31) = 26.4, *p*<.001, whereas there was no effect of matching nor an interaction, *F*s<1.

Mean RTs relative to the onset of the visual adjective in correct sentences were 1581 and 1587 ms when the auditory adjective of Task 2 semantically matched or mismatched, respectively. Mean RTs were only 1249 and 1211 ms for incorrect sentences when the auditory adjective of Task 2 matched or mismatched, respectively. An ANOVA yielded a significant main effect of Correctness, *F*(1,31) = 147.4, *p*<.001, but none of Matching, *F*(1,31) = 2.0, *p*>.1. Further, ANOVA revealed only a trend for an interaction of both factors, *F*(1,31) = 2.9, *p* = .097.

#### ERP Data

**Figure 4:**
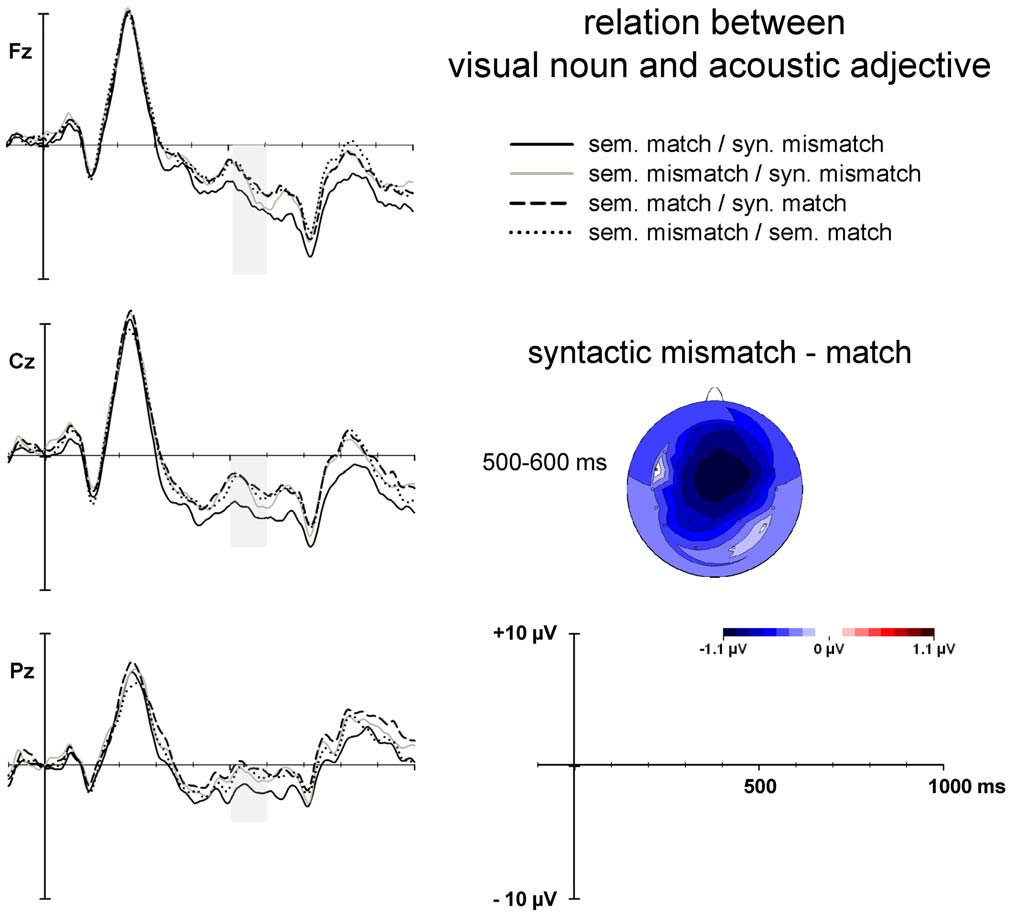
ERPs as a function of matching or mismatching between the visual noun of Task 1 and the acoustic adjective of Task 2, referred to a 200 ms- baseline prior the onset of these stimuli. *Left*. ERP waveforms at selected midline electrode sites. *Right*. Scalp distribution of the difference wave between syntactically mismatching minus matching pairs.

Again, mean ERP amplitudes were first analyzed referring to the initial baseline 200 ms prior to the onset of noun and acoustic adjective. In contrast to the semantic condition, a significant main effect of Correctness appeared within this interval, *F*(1,31) = 5.9, *p*<.05. In order to understand this effect it is important to remember that the manipulation of sentence correctness (correct syntactic relationship between noun and adjective within the sentence) causes converse relationships between the within-sentence noun and the extraneous adjective. That is, in correct sentences nouns and auditory adjectives are syntactically mismatching whereas in incorrect sentences nouns and auditory adjectives are syntactically matching (see Fig. 2). As shown in Figure 4, this syntactic mismatch between the acoustic adjective and the visual noun elicits an enhanced negativity, most pronounced over frontocentral electrodes and for the condition where both words were semantically related. Post-hoc analyses in consecutive 50-ms segments revealed that this negativity lasts until 350 ms after the onset of the visual adjective. Figure 4 shows the scalp distribution of the ERP difference wave between syntactically mismatching and matching acoustic adjective-noun pairs and the corresponding grand mean ERPs to all conditions.

Because of the mismatching effect at the time of adjective presentation, the pre-noun baseline was retained. As the effects due to the relation of the noun and the acoustic adjective do not last beyond 350 ms following the visual adjective onset, it appears save to use this early baseline. In order to be consistent with the results of the semantic conditions, we will consider the onset of the visual adjective as time zero also in the present condition.

**Figure 5:**
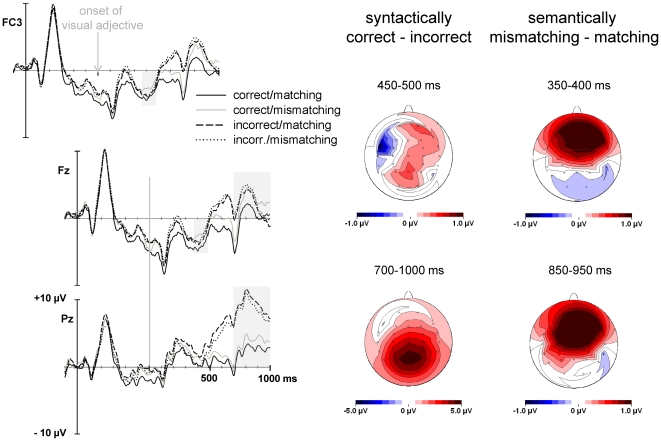
ERPs to syntactically correct and incorrect adjectives of Task 1, referred to the initial 100 ms baseline prior the noun. *Left*. ERP waveforms at selected electrode sites. Vertical grey lines mark the onset of the visual adjective of Task 1. *Middle*. Difference maps of the effects of syntactic violation in the LAN and P600 time windows. *Right*. Scalp distribution of ERP differences to semantically mismatching minus matching adjectives of Task 2.

Results of the ANOVA are summarized in Table 3. A first effect of Correctness appeared – in interaction with electrode site – between 450 and 500 ms, *F*(26,608) = 2.5, *p*<.05, *ε* = .166. Here, syntactically incorrect sentences elicited an enhanced negativity at left anterior electrodes, which is typical for the LAN (see Fig. 5). Between 700 and 1000 ms, ANOVAs revealed significant main effect of Correctness, *F*s(1,31)>6.5, *p*s<.05, as well as significant Electrode by Correctness interaction, *F*s(26,806)>20.1, *p*s<.001, .162<*ε*>.217. In contrast to the earlier effect, this effect of syntactic sentence correctness consists in an enhanced positivity over posterior electrode sites, corresponding to the P600.

**Table 3:** ANOVA results – syntactic condition.

Source	df	350–400	400–450	450–500	700–750	750–800	800–850	850–900	900–950	950–1000
Correctness (Corr.)	1,31			2.6*	64.5***	37.8***	35.2***	26.9***	13.3**	6.6*
Matching (Match.)	1,31									
Corr.*Match.	1,31	4.4*								
Corr.*Electrode (El.)	26,806			2.6*	20.1***	32.3***	30.9***	27.3***	25.4***	28.6***
				.262	.166	.187	.163	.183	.192	.216
Match.*El.	26,806	3.5**	4.8***	3.4**		2.3*		2.5*	4.8*	
		.218	.226	.262		.236		.219	.217	
Match.*Corr.*El.	26,806	2.7*								
		.208								

Note. *F*-values with *p* (***<.001, **<.01, *<.05) and *ε* for Greenhouse-Geisser correction. Only significant results are reported.

Between 350 and 500 ms, ANOVAs also yielded a significant interaction between the factors Matching and Electrode, *F*s(26,806)>3.4, *p*s<.01, .217<*ε*>.263, consisting of an anterior positivity to semantically mismatching relative to matching adjectives (see right panel of Figure 5). A topographically similar effect also appeared between 700 and 900 ms, *F*s(26,806)>2.3, *p*s<.05, .214<*ε*>.237. In addition, there were small interactions between sentence correctness and matching as well as with electrode (please, see Table 3). As can be seen in Figure 5, in late intervals these interactions are due to smaller frontal positivities to syntactically correct and matching adjectives relative to all other conditions (with one violation or mismatch, respectively, or a double violation). However, these interactions between the within-sentence violation and the mismatching with the extraneous information do not appear at the LAN or P600 components themselves.

## Discussion

The present study investigated whether the syntactic and semantic processing streams in sentence comprehension follow strict rules in a rather modular system or whether they are characterized by a more open and flexible nature. Thus, we studied whether semantic and syntactic processing is influenced by matching or mismatching information that had to be simultaneously held in auditory short-term memory. Complementing our previous study [30], the match of sentence-extraneous and sentence-internal information was not manipulated within but across the syntactic/semantic domain. That is, semantic within-sentence violations, which appeared between the visual adjective and the preceding noun, were combined with syntactic (across-stream) non-matches between the visual and the acoustic adjectives. Conversely, syntactic within-sentence violations were combined with semantic (across-stream) non-matches.

As expected, in both the semantic and syntactic conditions the typical ERP components were elicited by within-sentence violations. The present findings in the *semantic condition* replicate that within-sentence semantic violations elicit not only an N400 component, but also a P600, providing further evidence for the function of the P600 in the context of semantic processing (e.g., [1], [12], [30]). Although somewhat smaller in amplitude, this P600 component to semantic violations was rather similar to the one elicited by syntactic violations. Thus, the present data underline recent proposals suggesting that the P600 is not restricted to syntactic processing, but rather reflects a third, combinatorial stream (e.g., [20]). In addition to the P600, syntactic within-sentence violations elicited a LAN, indicating the effect of morphosyntactic violation in sentence comprehension (see further [37]).

Importantly, in the semantic condition neither the N400 nor the P600 were affected by syntactically mismatching sentence-extraneous information, that is, the syntactic information of the auditory adjective of Task 2 did not affect ERP components elicited by semantic violations within the sentences. This finding confirms our previous report that sentence-external syntactic information is not taken into consideration in sentence processing. It further extends it to the case where the external syntactic information is combined with a within-sentence semantic violation. This indicates that there is no interplay between within-sentence semantic processing and external syntactic information, which contrasts to at least some of the findings from combined within-sentence violations [1].

Similarly to the robustness of these ERP components to semantic within-sentence violations against sentence-extraneous syntactic mismatches, both the LAN and P600 to syntactic within-sentence violations (“syntactic condition”) were unaffected by semantic mismatching of the sentence-extraneous adjectives. This is in line with our previous findings, showing both components to syntactic within-sentence violations to be insensitive against sentence-extraneous mismatching information. Thus, the present study constitutes additional evidence for the robust algorithmic nature of the syntactic stream along the parsing processes, as syntactic violations are affected neither by syntactic [30] nor semantic (present study) sentence-extraneous information. Importantly, in the present study we found a P600 to both syntactic and semantic within-sentence violations, which was not affected by the sentence-extraneous information from the other domain. This was not the case in our previous study [30], where the P600 to semantic violations was affected by sentence-extraneous semantic manipulations. It is possible that syntactic violations yield maximal P600 values, over which external semantic mismatching cannot exert further influence.

Although there were no interactions between the violations and extra-sentential material in both domains, extraneous semantic information seemed to impact sentence processing, as indicated by an ERP effect to the acoustic, semantically non-matching adjectives in the syntactic condition. This effect of semantically mismatching extraneous adjectives consisted in a fronto-central positivity, yielding significant results from 350 to 500 ms and from 700 to 900 ms. A visual inspection of the data revealed that this fronto-central positivity extended along the 350–900 ms period. This fronto-central positivity to sentence-extraneous semantic mismatches resembles the frontal P600 or FP600, reported by others [38], [39], which is suggested to reflect ambiguity resolution and/or high discourse level complexity.

On the one hand, this effect conforms to our previous study by showing an influence of sentence-extraneous semantic information. On the other hand, it differs from our previous findings because the semantic mismatches with sentence-extraneous materials did not yield an N400 effect [30]. The emergence of a fronto-central positivity instead of a parietal negativity might be accounted for by methodological and procedural differences between the present and our previous study. As an attempt to avoid anticipatory processes with respect to the type of violation, a major modification of the experimental design concerned the randomized presentation of all conditions in the present study, whereas in the previous experiment semantic and syntactic violations had been manipulated block-wise.

In the present study, the fronto-central positivity appeared when semantically mismatching extraneous material concurred with semantically correct sentences (i.e., syntactic condition), introducing a new element into the semantic frame, possibly increasing the complexity of the discourse model of the sentence in question. Then, the randomized presentation of both the semantic and syntactic conditions within a given block may have increased the difficulty of the task as compared to our previous study, as might be reflected in much prolonged RTs and enhanced error rates in the present study. This increased task difficulty may have forced the subjects to adopt different strategies in order to deal with the sentence-extraneous semantic material. It is possible that under the less predictable conditions of the present study, sentence-extraneous information was included into discourse-related knowledge. In more predictable and therefore easier conditions of our previous study, sentence-extraneous information was taken into consideration, as demonstrated by the presence of the N400 component, but in a qualitatively different way as in the present study. Please note that already the previously observed N400 to sentence-external material had been topographically shifted towards more posterior sites as compared to the sentence-internal N400, possibly a first indication of a superposition with the frontal positivity observed here as a full-fledged component. It will be of interest for future research to delimit the conditions under which the parietal negativity of the N400 tilts into a fronto-central positivity. The fact that in the present experiment several types of combinations of sentences with extraneous material appeared twice as often as other combinations, as explained in the Procedure section, could not explain a FP600. Whatever the reasons for the here-observed ERP-effect of sentence-extraneous semantic information, on a more general level the present findings confirm our previous observation that semantic processing is open to external information even when this information is presented in a different modality.

A further finding deserves discussion. As described in the Methods section, the experimental manipulation of sentence correctness and mis/matching between the acoustic and the visual adjective also had consequences for the relationship between the acoustic adjective and the visual noun. In the syntactic condition, the acoustic adjective was semantically mismatching to the written adjective but to the preceding written *noun* it was *syntactically* mismatching. This mismatch had elicited an enhanced negativity over the vertex during the period prior to the written adjective. Although elicited by morphosyntactic mismatching, its distribution does not seem to reflect a LAN component. Instead, it resembles an N400-like modulation, similar to the one obtained for within-sentence violations in the semantic condition. Albeit rare, this finding is not unprecedented. For instance, an N400 to morphosyntactic (number) violations has recently been reported by Severens and colleagues [40], who interpreted this finding as reflecting semantic implausibility. The same interpretation may apply to our present findings, considering that this N400 emerged as the consequence of the morphosyntactic (gender) mismatch between two words. What makes the present results interesting is that these two words also differed not only in modality, but (and mainly) in their relevance for sentence processing as well.

In the present experiment we have been able to observe effects of the acoustic adjectives on both the ERPs to the visual nouns and adjectives of the sentences, even if the onsets of the acoustic adjectives and the written nouns were simultaneous whereas the written adjectives appeared always some time after the termination of the acoustic adjectives. Especially, the effect of the acoustic adjective may be subject to storage and decay within working memory. Therefore, the interplay between the different domains might depend also on the temporal relationship between the elements at work, a factor that has not been taken into consideration here.

In conclusion, the present results revealed that within-sentence processing of semantic violations is unaffected by extraneous syntactic manipulations whereas extraneous semantic manipulations did exert an influence on sentence processing. However, this influence of semantic information observed in the syntactic condition does not seem to directly affect the syntactic stream because the LAN, reflecting purely syntactic analyses, was entirely unaffected by semantic sentence-extraneous information. Thus, even though sentence-extraneous semantic information can penetrate sentence processing, it does not seem to interact with syntactic analyses proper. Together with the previous observation that only semantic, but not syntactic sentence-extraneous manipulations affect within-sentence processing of the same stream [30], the current findings underscore the open nature of semantic and the algorithmic nature of syntactic processing during sentence comprehension.
